# *Pantoea trifolii* sp. nov., a novel bacterium isolated from *Trifolium rubens* root nodules

**DOI:** 10.1038/s41598-024-53200-2

**Published:** 2024-02-01

**Authors:** Sylwia Wdowiak-Wróbel, Michał Kalita, Marta Palusińska-Szysz, Monika Marek-Kozaczuk, Wojciech Sokołowski, Teresa A. Coutinho

**Affiliations:** 1 Department of Genetics and Microbiology, Institute of Biological Sciences, Maria Curie-Skłodowska University, Akademicka 19, 20-033 Lublin, Poland; 2 Department of Biochemistry, Genetics and Microbiology, Centre for Microbial Ecology and Genomics/Forestry and Agricultural Biotechnology Institute, University of Pretoria, Pretoria, 0002 South Africa

**Keywords:** Bacteria, Soil microbiology

## Abstract

A novel bacterium, designated strain MMK2^T^, was isolated from a surface-sterilised root nodule of a *Trifolium rubens* plant growing in south-eastern Poland. Cells were Gram negative, non-spore forming and rod shaped. The strain had the highest 16S rRNA gene sequence similarity with *P. endophytica* (99.4%), *P. leporis* (99.4%) *P. rwandensis* (98.8%) and *P. rodasii* (98.45%). Phylogenomic analysis clearly showed that strain MMK2^T^ and an additional strain, MMK3, should reside in the genus *Pantoea* and that they were most closely related to *P. endophytica* and *P. leporis*. Genome comparisons showed that the novel strain shared 82.96–93.50% average nucleotide identity and 26.2–53. 2% digital DNA:DNA hybridization with closely related species. Both strains produced siderophores and were able to solubilise phosphates. The MMK2^T^ strain was also able to produce indole-3-acetic acid. The tested strains differed in their antimicrobial activity, but both were able to inhibit the growth of *Sclerotinia sclerotiorum* 10Ss01. Based on the results of the phenotypic, phylogenomic, genomic and chemotaxonomic analyses, strains MMK2^T^ and MMK3 belong to a novel species in the genus *Pantoea* for which the name *Pantoea trifolii* sp. nov. is proposed with the type strain MMK2^T^ (= DSM 115063^T^ = LMG 33049^T^).

## Introduction

The genus *Pantoea* was first described by Gavini et al. in 1989^1^ and it belongs to the family *Enterobacteriaceae*, order Enterobacteriales and phylum Pseudomonatoda. The genus contains 23 validly published species^2^ isolated from various ecological niches including soil^3^, water^4^, clinical samples^5^ and plant material^6^. They have been isolated as plant pathogens^7^, endophytes^6^, biocontrol agents^8^, plant growth promoters^9^ and as bioremediation agents^10^.

Certain species of *Pantoea* are well-known plant pathogens. For example, *P. stewartii* subsp. *stewartii*, is responsible for the development of Stewart's vascular wilt disease in sweet corn and maize^11^. *Pantoea agglomerans* pv. *gypsophilae* causes crown and root gall disease in gypsophila, whereas *P. agglomerans* pv. *betae* infects beets^12^. *Pantoea ananatis* causes a range of disease symptoms in different hosts including onions, rice, maize and melon^13^.

Some *Pantoea* species have been reported as causing opportunistic infections in humans^14^. Infection occurs mainly through wounding of the skin by plant material. A hospital-acquired infection caused by these species has also been described, primarily in immunocompromised patients. *P. agglomerans* infection may lead to skin allergies, septic arthritis, or synovitis. Strains of this species also causes periostitis, endocarditis, osteomyelitis, and sepsis^12,15^. However, it should be noted that some reported cases of *P. agglomerans* infections in humans are examples of pathogen misidentification^16^.

Strains of some *Pantoea* species have been described as epi- or endophytic plant symbionts^17^ while others have found application in industry. For instance, antimicrobials produced by some representatives of the genus, for example, *P. agglomerans,* are used as biological control agents in commercial products, such as BlightBan C9-1 and Bloomtime Biological to fight fire blight in apple and pear orchards^18,19^. It has been shown that some strains of *P. agglomerans* can induce systemic acquired resistance in some plants^20^. *P. ananatis* strains have also been shown to produce antifungal and antibacterial compounds, for example, phenazines and pantocins, and can inhibit plant infections caused by such phytopathogens as *Penicillium expansum* and *Botrytis cinerea*^21–23^. *Pantoea* species have been shown to contribute to increased biomass production in a variety of plants through, for example, the synthesis of phytohormones such as indole-3-acetic acid, gibberellic acid, and siderophores or the production of carotenoids^17^.

This paper describes a new endophytic bacterium isolated from root nodules of *Trifolium rubens* collected in south-eastern Poland. This putative beneficial, culturable strain was characterized using a polyphasic approach which included phenotypic, chemotaxonomic and genomic analyses.

## Materials and methods

### Isolation of bacterial strains

Strains were isolated from root nodules of *Trifolium rubens*, a plant growing in the south- eastern region of Poland (51.27454 N, 23.35748 E). Nodules were harvested and surface-sterilized by rinsing them several times with sterile water and placing them in 0.1% HgCl_2_ for three minutes. Thereafter, they were rinsed twice with sterile water, immersed in 70% ethanol for 2 min, and finally rinsed thrice with sterile water. The nodules were crushed in 0.5 ml of sterile saline solution (0.85% NaCl in MilliQ water) with sterile forceps, the suspension was diluted tenfold and 100 µl of the dilution was streaked on to yeast extract-mannitol (YEM) agar plates and incubated at 28 °C for three to four days^24^. The YEM medium was composed of the following ingredients (in 1000 ml distilled water): yeast extract 1.0 g, mannitol 10.0 g, dipotassium phosphate 0.5 g, magnesium sulphate 0.2 g, sodium chloride 0.1 g, calcium carbonate 1.0 g (pH 6.8 ± 0.2). After incubation, the single bacterial colonies were picked and further purified by repeated streaking to obtain pure cultures. Purified bacterial cultures were maintained on YEM agar slants at 4 °C as well as at − 70 °C in YEM broth with 15% (v/v) glycerol.

### Research involving plants

Procedures involving the collection of plant material were carried out in accordance with institutional, national and international rules and legislation. *Trifolium rubens* is not included in the list of protected and endangered species of wild plants in Poland. Therefore, no permissions were required for the collection of research material. The species *Trifolium rubens* was identified by Dr. Mykhaylo Chernetskyy from the Maria Curie-Skłodowska University Botanical Garden in Lublin. To identify *Trifolium rubens*, the characteristic features of the species as described in the literature was used^25,26^.

### Phenotypic and chemotaxonomic analyses

Gram stain reaction was performed using standard methods. The slides were examined using the 100 × oil immersion objective using an Olympus CX23 microscope (Olympus, Japan). The Schaeffer–Fulton staining technique was used to determine the presence of spores in the tested strains^27^. The slides were observed under the same microscope as for the Gram stain method. Growth tests were performed in YEM broth at 28 °C. The growth temperature range was tested on the same medium at 15, 28, 37, 42 and 45 °C for 4–8 days. Tolerance to NaCl was determined based on the growth of the bacteria on YEM agar supplemented with 8, 9 and 10% NaCl (w/v) concentrations and incubated 28 °C for four to eight days. The pH range for growth was established by incubating the strain in YEM broth at pH levels ranging from 5.0 to 10.0 at intervals of 1 pH unit. The Biolog GEN III system (Biolog Inc. Hayward, CA, USA) and GN A + B-ID system (Microgen) were used in accordance to the manufacturer’s recommendations. Enzymatic characterization of the bacterial isolates was performed using API ZYM strips (BioMérieux, France) according to the manufacturer’s protocol. Catalase and oxidase activity was determined using standard methods. Cellular fatty acids in the form of their methyl esters were prepared according to the protocol of Wollenweber and Rietschel^28^ and analysed by using an Agilent Technologies (Instrument 7890) gas chromatograph connected to a mass selective detector (Agilent Technologies MSD5975C, inert XL EI/CI) (GLC-MS), using helium as a carrier. The components of fatty acid methyl ester were determined mainly by their chromatographic and mass spectral characteristics. The positions of the branching methyl group, cyclopropane ring, and the double bonds were determined by an analysis of mass spectra of fatty acid pyrrolidines^29^. Each fatty acid was quantified by calculating its peak area relative to the total peak area^30^.

### In vitro assessment of plant growth promoting characteristics

Indole-3-acetic acid (IAA) production in *Pantoea* strains was detected using a qualitative test. The IAA production was determined using Salkowski reagent as described by Luziatelli et al.^31^.

The indole production was detected in M9 broth medium^32^ with 0.5 mM of l-tryptophan as described by Gnat et al.^33^.

Assessment of HCN production by *Pantoea* strains was carried out by using the method described by Lorck^34^.

The ability of *Pantoea* strains to solubilize phosphate was determined on Pikovskaya agar^35^. The bacterial strains were streaked on Pikovskaya agar and incubated at 28 °C for 10 days. Formation of a transparent halo around the colony indicates solubilisation of phosphate.

Chrome azurol S medium (CAS, Sigma-Aldrich, USA) was used to test the capability of the microorganisms to produce siderophores^36^. The strains were spotted on CAS medium and inoculated for three to four days at 28 °C. Formation of a coloured halo around the colony indicated siderophore production.

Cellulase activity of studied strains were detected on carboxymethylcellulose (CMC) media according to the method described by Kasana et al.^37^. Gram’s iodine forms a bluish-black complex with cellulose. The positive reaction for cellulase production is visible as a sharp and distinct zone around the microbial colonies^37^.

Proteolytic activity was evaluated in nutrient agar supplemented with 10% skim milk^38^. *Pantoea* strains were spot inoculated and incubated for 48–72 h at 28 °C. The enzymatic degradation of milk protein was visible as a clear zone around a bacterial colony. All the experiments described above were performed in triplicate.

### In vitro assessment of antifungal and antibacterial activity

The antagonism test was performed against four phytopathogenic fungi listed in Table 1. Fungal strains were grown on a potato dextrose agar (PDA, Biomaxima, Poland) plates for 7 days. A 5-mm agar plug of mycelium was excised with a sterilized cork borer and was placed in the centre of a YEM agar plate inoculated with the *Pantoea* strain. The interactions of the tested strains with fungal pathogens were examined every day for a period of three weeks. Three biological replications were performed for each treatment. The control were plates inoculated only with the fungal pathogen. The plates were assessed as follows: (−) no effect on inhibiting the growth of the fungal phytopathogen; (+) inhibition of the growth of the fungal phytopathogen compared to the control.

**Table 1:** Bacterial and fungal strains used in in vitro antimicrobial activity tests.

Strain	Culture media and growth condition (°C)
*Xanthomonas vesicatoria* NCCB 92,059	LB, 28
*Agrobacterium fabrum* C58	LB, 28
*Pseudomonas syringae* pv. *syringae* 2905	LB, 28
*Erwinia amylovora* 659	LB, 28
*Diaporthe rudis* CBS 109,492	PDA, 25
*Fusarium oxysporum* 10Fo01	PDA, 25
*Botrytis cinerea* 10Bc01	PDA, 25
*Sclerotinia sclerotiorum* 10Ss01	PDA, 25

The studied *Pantoea* strains were tested for their ability to inhibit the growth of four phytopathogenic bacteria listed in Table 1. using the agar plug diffusion method^39^. *Pantoea* strains were cultured on YEM agar for five days at 28 °C. After this time, 9 mm agar discs with the strain were cut out using a sterilized cork borer. An agar disc with the tested strain was placed at the centre of the LB agar plates (Biomaxima, Poland) inoculated with 24 h cultures of the phytopathogens in LB broth (Biomaxima, Poland) (10^8^ CFU/ml). The plates were incubated at 28 °C and strain interactions were checked after 24, 48 and 72 h. The appearance of a zone of inhibition of the growth of phytopathogens around bacterial colonies was considered a positive result.

### 16S rRNA gene phylogenetic analysis

The genomic DNA was extracted using a bacterial genomic DNA extraction kit (GeneMATRIX Tissue & Bacterial DNA Purification Kit, EURx). The 16S rRNA gene was amplified and sequenced with the bacterial universal primers fD1 and rD1 described by Weisburg et al.^40^. PCR amplification reactions were carried out with ReadyMix™ Taq PCR Reaction Mix (Sigma) according to the manufacturer’s recommendations. The amplified products were purified with Clean-Up purification columns (A&A Biotechnology) and sequenced with BigDye Terminator Cycle sequencing kit using the 3500 Genetic Analyzer according to the manufacturer’s procedures (Life Technologies) as described elsewehere^41^. The 1337 bp long 16S rRNA gene sequence fragments of MMK2^T^ and MMK3 were deposited in GenBank under the accession numbers OQ799602 and OQ799603. The sequence similarity searches were performed by using the BLAST algorithm. Phylogenetic tree based on 16S rRNA gene sequences were constructed using the software MEGA X^42^ and the maximum likelihood algorithm with Tamura 3-parameter model^43^. Bootstrap values were derived from 1000 replications.

### Genome sequencing, annotation and phylogenomic analyses

The whole genome of strains MMK2^T^ and MMK3 were sequenced on a PacBio platform by Inqaba Biotechical Industries (Pty) Ltd (Pretoria, South Africa) and assembled using the SMRTLink v. 11.0 software (PacBio). Genome sequences of the type strain MMK2^T^ and strain MMK3 were deposited in GenBank database under the accession numbers JANIET000000000.1 and JANIES000000000.1, respectively. Genome annotation was carried out automatically with the Genome Annotation Service using the RAST tool kit^44^, available at BV-BRC web resources^45^. The antiSMASH tool was used to identify putative biosynthetic gene clusters in the analysed genomes^46^. The genomes of the type strains of all other *Pantoea* species were obtained from GenBank. To determine the relationship between MMK2^T^, MMK3 and closely related species, phylogenomic analyses were performed using the bacterial Phylogenetic Tree Service available at BV-BRC web resources^45^ and utilizing the codon tree method and the RAxML program^47^. The average nucleotide identity (ANI) and digital DNA:DNA hybridization (dDDH) relatedness between MMK2^T^, MMK3 and types strains of closely related species were calculated using the ANI Calculator^48^ and the Genome-to-Genome Distance Calculator^49^, respectively.

## Results and discussion

### Isolation, morphology, physiology and biochemical features of *Pantoea trifolii*

The root nodules of legumes are inhabited mainly by bacteria capable of fixing atmospheric nitrogen as part of a symbiotic relationship with the host plant. The nitrogen-fixing symbiotic bacteria, collectively named as rhizobia, belong to different genera of *Alpha*- (e.g. *Bradyrhizobium*, *Ensifer*, *Mesorhizobium*, *Rhizobium*) and *Betaproteobacteria* (e.g. *Paraburkholderia*, *Cupriavidus*) classes^50^. In addition to the typical rhizobia, many other bacteria are often isolated from sterile root nodules, some of which are unable to fix nitrogen or induce nodulation. They are called nodule endophytes or nodule-associated bacteria^51^. Many of these nodule endophytes may be beneficial to plants due to their plant growth promoting effects^52^.

In our study, we isolated bacteria from sterile root nodules of *Trifolium rubens* plants growing in south-eastern Poland. Most of the examined root nodules were colonized by bacteria identified as *Rhizobium* spp. (data not shown), but two nodules collected from two different plants were also inhabited by bacteria that formed yellow colonies and produced a blue-violet pigment on YEM agar plates. These two isolates, designated MMK2 and MMK3, were used for further analyses.

The cells of MMK2^T^ and MMK3 are Gram negative, non-spore forming rods. The strains are able to grow at 37 °C, but not at 42 °C (optimum, 28 °C), at a pH in the range of 5.0–9.0 and in the presence of up to 9% NaCl, but not 10%. The catalase test was positive while the oxidase test was negative for both strains. Both strains form yellow colonies on the YEM agar and produce a blue-violet pigment that diffuses into the medium. To date, only a single strain of *Pantoea agglomerans* has been shown to produce a blue pigment^53^. The MMK2^T^ strain utilized dextrin, d-maltose, d-trehalose, d-cellobiose, gentiobiose, β-methyl-d-glucoside, d-salicin, *N*-acetyl-d-gucosamine, *N*-acetyl-β-d-mannosamine, α-d-glucose, d-mannose, d-fructose, d-galactose, 3-methyl glucose, d-fucose, l-fucose, l-rhamnose, inosine, d-sorbitol, d-mannitol, d-arbitol, myo-inositol, glycerol, d-glucose-6-PO_4_,d-fructose-6-PO_4_, d-galacturonic acid, l-galactonic acid lactone, d-gluconic acid, d-glucuronic acid, glucuronamide, mucic acid and d-saccharic acid, l-lactic acid, citric acid, d-malic acid, l-malic acid, bromo-succinic acid, γ-amino-butyric acid, acetoacetic acid, acetic acid and formic acid. The strain also used glycyl-l-proline, l-alanine, l-arginine, l-apartic acid, l-glutamic acid, l-histidine and l-serine. Negative reactions were recorded for d-turanose, stachyose, d-raffinose, α-d-lactose, d-mellibiose, *N*-acetyl neuraminic acid, d-aspartic acid, d-serine, gelatin, l-pyroglutamic acid, pectin, quinic acid, *p*-hydroxy-phenylacetic acid, methyl pyruvate, d-lactic acid methyl ester, α-keto-glutaric acid, tween 40, α-hydroksy-butyric acid, α-d,l-butyric acid, propionic acid, and minocycline. Positive enzyme activities were noted for alkaline phosphatase, esterase (C4), esterase lipase (C8), acid phospatase, naphthol-AS BI-phosphohydrolase, β-galactosidase, β-glucosidase and *N*-acetyl-β-glucosaminidase but not for lipase (C14), leucine arylamidase, valine arylamidase, cystine arylamidase, trypsin, α-chymotripsin, α-galactosidase, β-glucuronidase, α-glucosidase, α-mannosidase or α-fucosidase. Strain MMK2^T^ and MMK3 could be clearly distinguished from closely related *Pantoea* species, *P. endophytica* 596^T^, *P. rodasii* DSM 26611^T^ and *P. rwandensis* DSM 105076^T^ (Table 2)^6,54^.

**Table 2:** Differential characteristics between MMK2^T^, MMK3, *Pantoea endophytica* 596^T^, *P. rodasii* DSM 26611^T^ and *P. rwandensis* DSM 105076^T^^6,54^.

	MMK2^T^	MMK3	*P. endophytica* 596^T^	*P. rodasii* DSM 26611^T^	*P. rwandensis* DSM 105076^T^
Oxidation of (Biolog GENIII):					
Dextrin	+	−	−	+	+
d-maltose	+	+	+	−	+
d-trehalose	+	+	+	−	+
d-cellobiose	+	+	−	−	+
Gentiobiose	+	+	+	−	+
Sucrose	−	−	−	−	+
β-methyl-d-glucoside	+	+	+	−	+
d-salicin	+	+	+	−	+
*N*-acetyl-d-glucosamine	+	+	+	−	+
*N*-acetyl-β-d-mannosamine	+	+	+	−	+
*N*-acetyl-d-galactosamine	−	−	−	−	+
α-d-glucose	+	+	+	−	+
d-mannose	+	+	−	−	+
d-fructose	+	+	−	−	−
l-rhamnose	+	+	+	−	+
Inosine	+	+	−	−	−
d-sorbitol	+	+	−	−	+
d-mannitol	+	+	+	−	+
d-arabitol	+	+	+	−	+
Myo-inositol	+	+	+	+	−
d-glucose-6-PO4	+	+	−	−	+
Troleandomycin	+	+	+	−	+
l-alanine	+	+	+	−	+
l-arginine	+	+	+	−	−
l-aspartic acid	+	+	+	−	+
l-glutamic acid	+	+	+	−	+
l-histidine	+	+	+	−	−
l-serine	+	+	−	−	+
Lincomycin	+	+	+	−	+
Pectin	−	−	−	+	+
Glucuronamide	+	+	−	+	−
Mucic acid	+	+	+	−	+
Quinic acid	−	+	−	+	−
d-saccharic acid	+	+	+	−	+
Methyl pyruvate	+	+	+	−	+
l-lactic acid	+	+	+	+	+
Citric acid	+	+	+	−	+
Bromo-succinic acid	+	+	−	−	−
Lithium chloride	+	+	+	−	+
γ-amino-butryric acid	+	−	+	+	−
Acetic acid	+	+	+	+	−
Aztreonam	+	+	−	−	+
Enzyme production (API ZYM):					
Esterase (C4)	+	+	+	+	( +)
Esterase lipase (C8)	+	+	+	+	( +)
Lipase (C14)	−	−	+	−	−
Valine arylamidase	−	−	−	(+)	+
Cystine arylamidase	−	−	−	−	+
Trypsin	−	−	−	−	+
β-galactosidase	+	−	−	+	+

− negative, + positive, (+) weak positive; in the case of the API ZYM test, the differences between + and (+) concern the color intensity.

The results of the analysis of cellular fatty acids in the form of their methyl esters are presented in Table 3. The major fatty acids of MMK2^T^ and MMK3 strains were C_16:0_ (44%), C_17:0_ cyclo (17%), and C_14:0_ 3-OH (16%). The strains were also characterised by the presence of C_18:1_ɷ13, C_16:1_ and C_19:1_, an unsaturated fatty acids and C_14:0_, C_18:0_, and C_20:0_ saturated fatty acids. The MMK3 strain produced 2% and 2.5% higher amounts of C_17:0_ cyclo and C_19:1_ɷ9, respectively, and 1.5% lower amounts of C_20:0_ compared to MMK2^T^. These strains differed from their closest relatives, *P. endophytica* and *P. leporis*, in the amounts of C_16:0_, C_17:0_ cyclo, and the presence of C_14:0_ 3-OH, C_19:1_, C_20:0_,^6,55^.

**Table 3:** Cellular fatty acid profiles of MMK2^T^ and MMK3.

Retention time (min)	Fatty acid	Relative content (%)	
		MMK2^T^	MMK3
6.85	12:0	1 ± 0.1	1 ± 0.1
11.15	14:0	3 ± 0.5	4.5 ± 0.1
14.96	16:1	4 ± 0.2	2 ± 0.2
15.41	16:0	44 ± 1	43 ± 0.6
15.57	3-OH 14:0	16 ± 1	15 ± 0.9
17.08	Cyclopropyl17:0	17 ± 0.5	19 ± 0.7
18.87	18:1ɷ^13^	6.5 ± 0.3	6 ± 0.6
19.23	18:0	4 ± 0.6	3 ± 0.3
19.35	i19:1	1 ± 0.3	2 ± 0.2
20.36	a19:1	1 ± 0.4	1 ± 0.1
20.81	n19:1ɷ^9^	0.5 ± 0.1	3 ± 0.8
22.34	20:0	2	0.5 ± 0.1

*a*, methyl branch at the anteiso carbon atom; *i*, methyl branch at the iso carbon atom, n, unbranched acid; cyclopropyl, cyclopropane ring structure.

### 16S rRNA gene phylogeny

**Figure 1:**
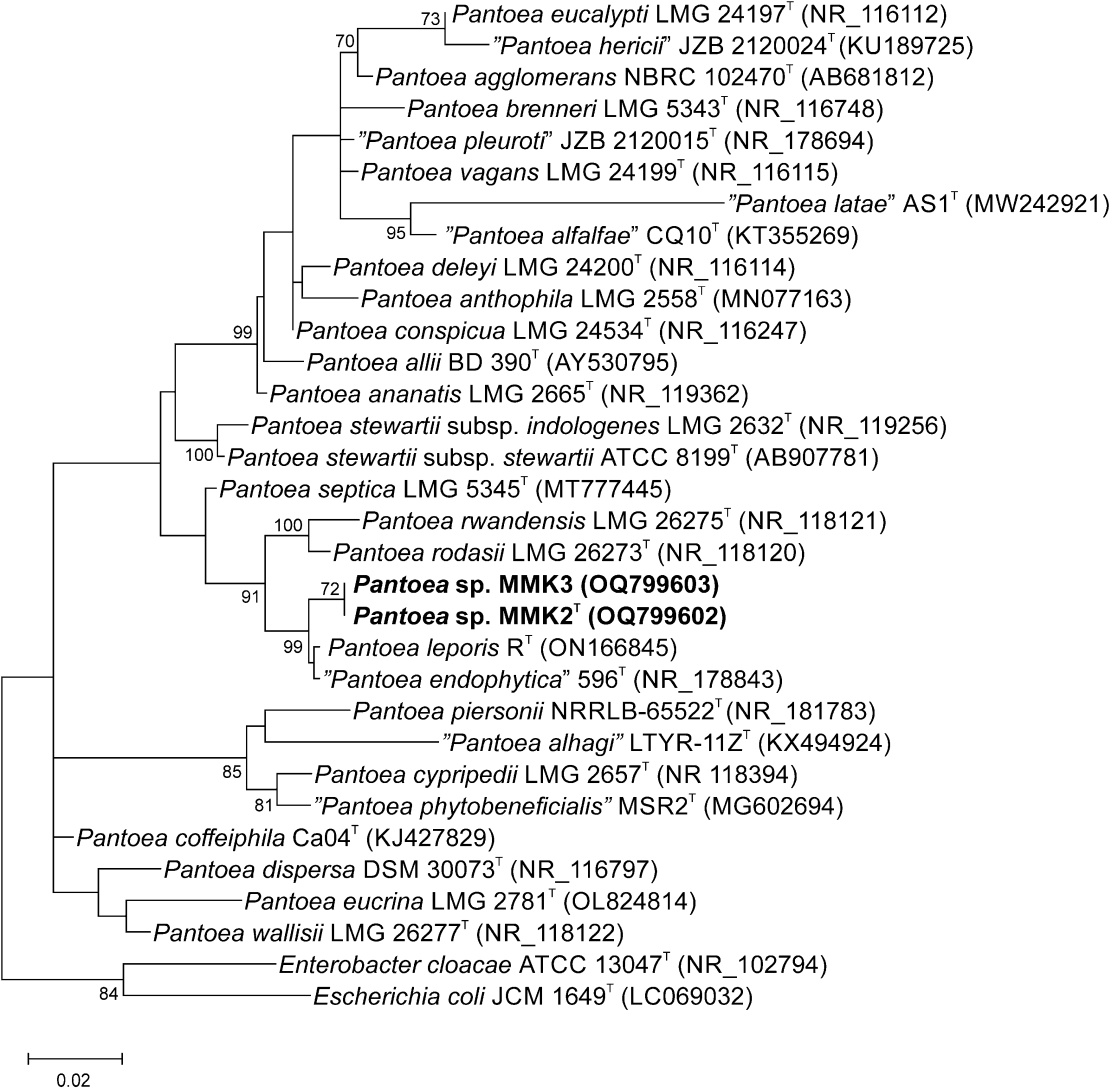
Maximum likelihood phylogenetic tree based on 16S rDNA sequences showing the relationship between MMK2^T^, MMK3 and type species of *Pantoea*. *Escherichia coli* JCM1649^T^ and *Enterobacter cloacae* ATCC 13047^T^ were used as outgroups. Species names written in quotation marks are not validly published (https://lpsn.dsmz.de/genus/pantoea). Bootstrap values greater than or equal to 70% are given at the branching points. The bar, 0.02, represents the number of substitutions per nucleotide.

Using the results of the phylogenetic analysis of the 16S rRNA gene encoding sequences, we were able to estimate the taxonomic position of the studied strains at the genus level. Phylogenetic analysis based on the maximum likelihood algorithm shows that strains MMK2^T^ and MMK3 belong to the genus *Pantoea* and have the highest 16S rRNA gene similarity to *P. endophytica* (99.4%) and *P. leporis* (99.4%), followed by *P. rwandensis* (98.8%) and *P. rodasii* (98.4%), with which the studied strains clustered together, but on a distinct branch in the phylogenetic tree (Fig. 1).

### Phylogenomic and genomic analyses

**Figure 2:**
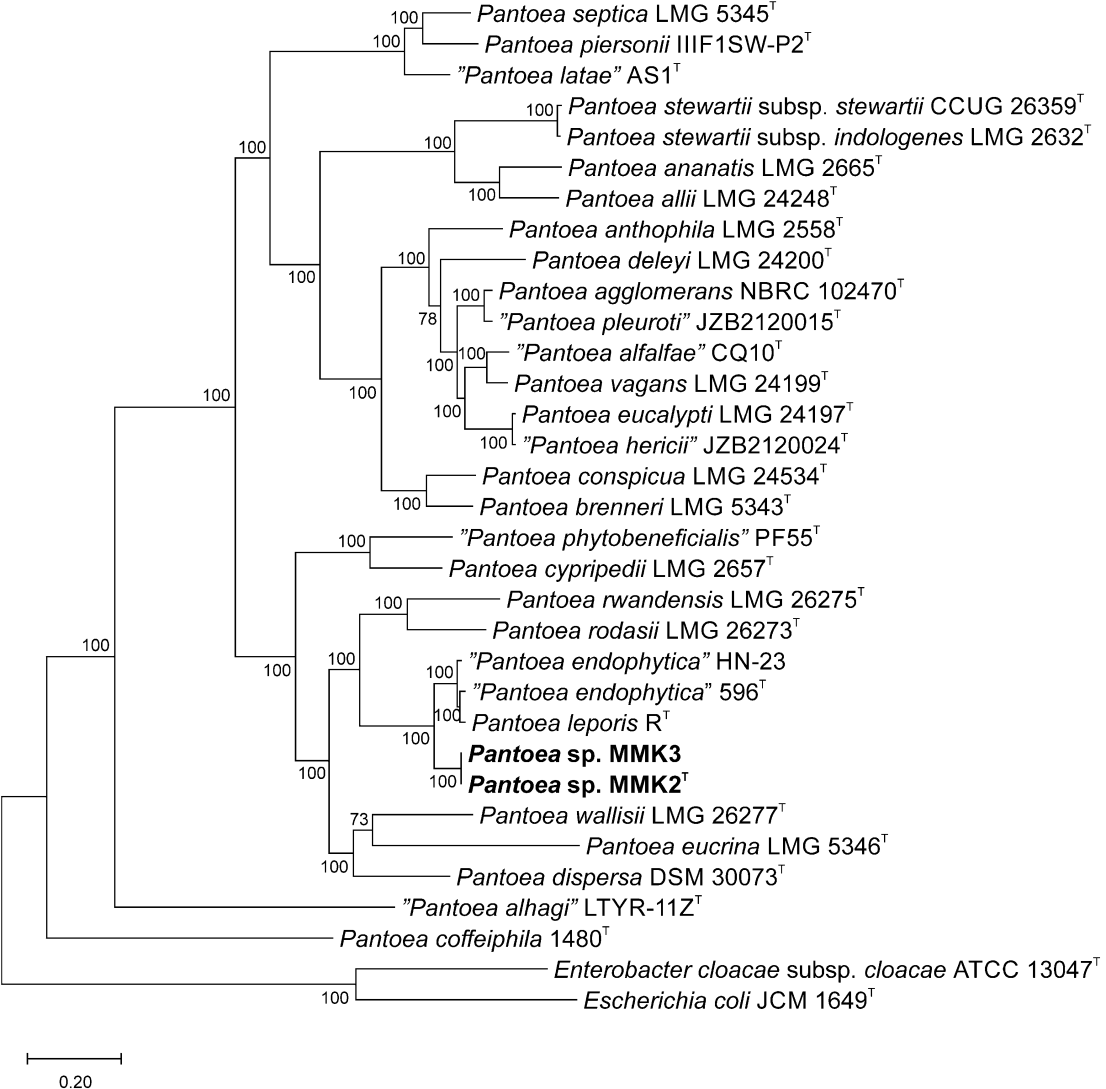
Phylogenomic tree based on 500 single-copy genes showing the relationship between strains MMK2T, MMK3 and *Pantoea* type strains. *Escherichia coli* JCM1649^T^ and *Enterobacter cloacae* ATCC 13047^T^ were used as outgroups. Species names written in quotation marks are not validly published (https://lpsn.dsmz.de/genus/pantoea). The branching points are labelled with bootstrap values. The bar, 0.20, represents the number of substitutions per site. The tree was generated using RAxML program.

The phylogenomic analysis based on 500 single-copy genes found in the genomes of *Pantoea* sp. MMK2^T^, *Pantoea* sp. MMK3 and reference strains clearly showed that MMK2^T^ and MMK3 were members of the genus *Pantoea*, and were most closely related to *P. endophytica* and *P. leporis* (Fig. 2).

The genome-derived ANI and dDDH values between MMK2^T^, and its closely related species were between 82.96 and 93.50% (Table 4) and 26.2–53.2% (Table 5), respectively, which is all below the threshold values of 95–96% ANI and 70% dDDH, i.e. recommended cut-off values for prokaryotic species delineation^56^.

**Table 4:** OrthoANI percentages between MMK2^T^, MMK3 and closely related *Pantoea* species.

	MMK2^T^	MMK3	*Pantoea endophytica* 596^T^	*Pantoea endophytica* HN-23	*Pantoea leporis* R^T^	*Pantoea rodasii* LMG 26273^T^	*Pantoea rwandensis* LMG 26275^T^
MMK2^T^	–						
MMK3	99.98	–					
*Pantoea endophytica* 596^T^	93.46	93.42	–				
*Pantoea endophytica* HN-23	93.35	93.50	98.24	–			
*Pantoea leporis* R^T^	93.42	93.43	98.56	98.28	–		
*Pantoea rodasii* LMG 26273^T^	83.95	83.87	83.83	83.89	83.82	–	
*Pantoea rwandensis* LMG 26275^T^	83.06	82.96	82.98	82.98	82.89	85.52	–

**Table 5:** dDDH percentages between MMK2^T^, MMK3 and closely related *Pantoea* species.

	MMK2^T^	MMK3	*Pantoea endophytica* 596^T^	*Pantoea endophytica* HN-23	*Pantoea leporis* R^T^	*Pantoea rodasii LMG* 26273^T^	*Pantoea rwandensis* LMG 26275^T^
MMK2^T^	–						
MMK3	100	–					
*Pantoea endophytica* 596^T^	53.1	53.1	–				
*Pantoea endophytica* HN-23	53.1	53.1	85.6	–			
*Pantoea leporis* R^T^	53.2	53.2	88.8	85.8	–		
*Pantoea rodasii LMG* 26273^T^	27.7	27.7	27.5	27.4	27.5	–	
*Pantoea rwandensis* LMG 26275^T^	26.5	26.5	26.2	26.3	26.2	29.9	–

**Figure 3:**
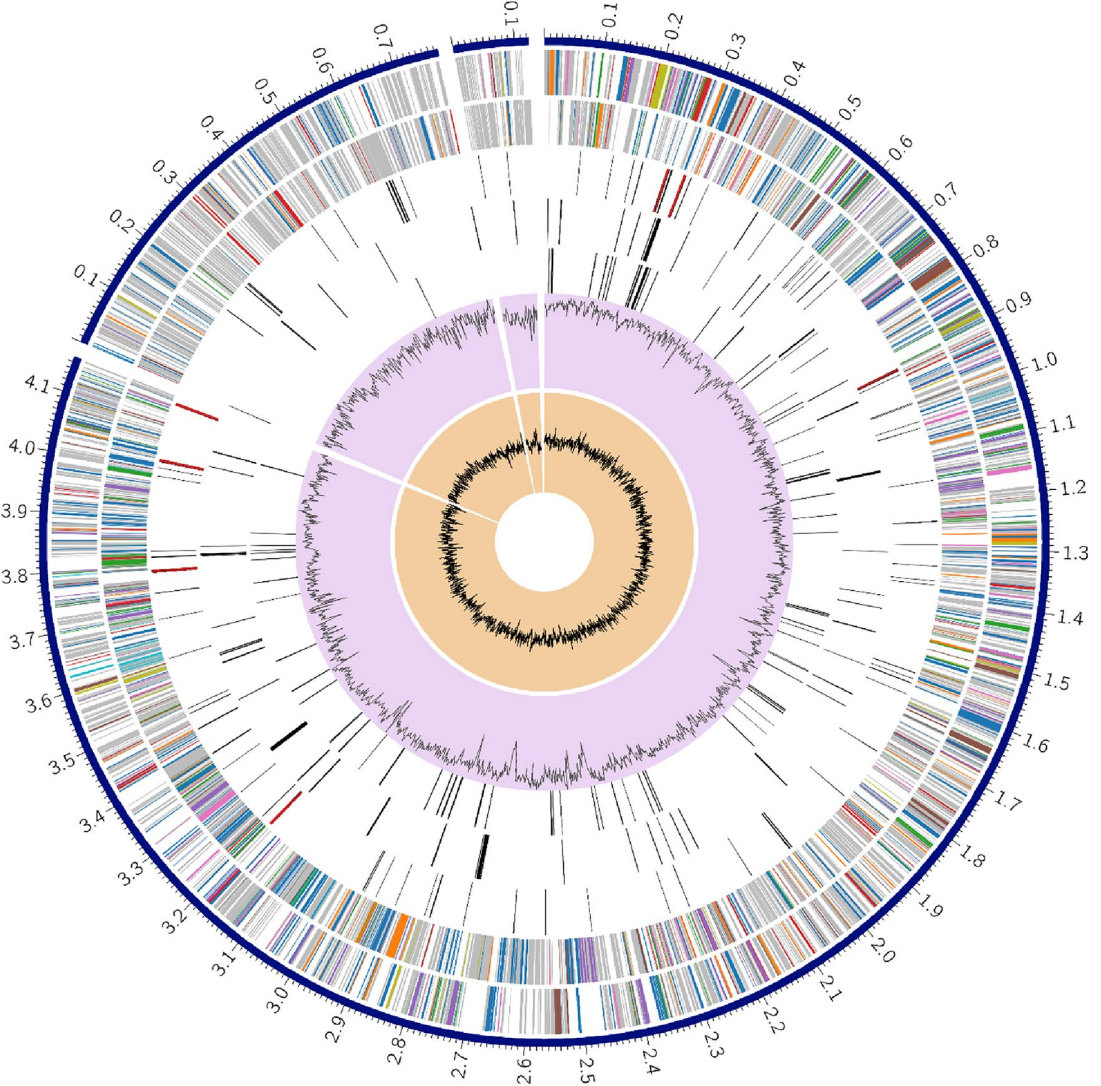
A circular diagram representing the genome of *Pantoea* MMK2^T^. The three contigs and their sizes are shown as the outermost ring. Coding sequences (CDS) on the forward strand, CDS on the reverse strand are represented by the second and third rings. The remaining rings represent RNA genes, CDS with homology to known antimicrobial resistance genes, CDS with homology to known virulence factors, guanine and cytosine (GC) content and GC skew.

The draft genome of strain MMK2^T^ was 5.06 Mb long and composed of 3 contigs with a N50 of 4.16 Mb and L50 of 1 and genome coverage of 560x (Fig. 3). The genome size is above the median being 4.85 Mb for the sequenced *Pantoea* strains^2^. It has genomic DNA G + C content of 54.63 mol % which is within the G + C content range of the genus *Pantoea*^5^. The MMK2^T^ genome contains 4721 protein coding sequences (CDS), 78 transfer RNA (tRNA) genes, and 22 ribosomal RNA (rRNA) genes. The annotation included 733 hypothetical proteins and 3988 proteins with functional assignments (Table 6). Among the proteins with functional assignments 1241 represented proteins with Enzyme Commission (EC) numbers, 1,030 belonged to proteins with Gene Ontology (GO) assignments, and 896 included proteins that were mapped to KEGG pathways^57^.

**Table 6:** Major protein coding genes of *Pantoea* MMK2^T^ and their distribution by subsystem category as annotated by the PATRIC annotation server.

Subsystem	Number of genes
Metabolism	937
Protein processing	250
Stress response, defense, virulence	184
Energy	272
Membrane transport	167
Cellular processes	153
RNA processing	75
Cell envelope	72
Regulation and cell signaling	25

### Analysis of biosynthetic gene clusters

Analysis of the MMK2^T^ and MMK3 genomes using the antiSMASH tool revealed the presence of two biosynthetic gene clusters (BGC) associated with the pigments production, i.e. carotenoids and aryl polyenes (APE). The aryl polyene gene cluster showed 94% similarity to the secondary metabolite BGC from *Xenorhabdus doucetiae*. In addition, a cluster of genes involved in the synthesis of carotenoids was found in the genomes of studied strains. This gene cluster from strains MMK2^T^ and MMK3 has the classical organization *crtEXYIBZ*, having all the necessary carotenoid synthesis genes for zeaxanthin glucosides^58^. The role of carotenoids in bacteria is related to the protection of the cell against stress factors, such as the toxic effects of reactive oxygen species (ROS), desiccation or salinity. They also act as photoprotectants against UV radiation (especially in the range from 320 to 400 nm). It was found that carotenoids can regulate membrane fluidity and participate in the organization of membrane domains^59^. The ability to synthesise carotenoids has been described for both pathogenic and endophytic members of the genus *Pantoea*. They have been found in *P. agglomerans*, *P. ananatis* and in the strain *Pantoea* sp. YR343^60–62^.

No gene clusters associated with the synthesis of the blue or violet pigment were identified in the MMK2^T^ and MMK3 genomes. The planned research on the analysis of the structure and function of the pigment may allow the identification of genes involved in its production. In addition to the gene clusters associated with pigment production, antiSMASH found BGC involved in the synthesis of frederiksenibactin, a triscatechol siderophore, in the genomes of the studied strains. Siderophores are compounds of low molecular weight and are characterized by high affinity to metal ions, mainly ferric, but also to ions, for example, aluminium, copper, cadmium or lead^63^. In the case of plant growth promoting bacteria (PGPB), the production of siderophores can positively influence the physiological and biochemical processes taking place in the host. In addition, through competition for iron ions, they can limit the development of phytopathogens in the environment^64^.

Frederiksenibactin is produced by an opportunistic human pathogen *Yersinia frederiksenii*^65^ but the BGC related to the synthesis of this siderophore was also identified by antiSMASH in the genomes of other *Pantoea* species, for example, *P. endophytica*, *P. rodassi* but not in the genome of *P. rwandensis*.

### In vitro plant growth promoting and antimicrobial characteristics of *Pantoea trifolii*

Among the tested traits related to the promotion of plant growth, both studied strains showed the ability to solubilize phosphates and produce siderophores. Additionally, strain MMK2^T^ was positive for the production of indole-3-acetic acid (IAA). Using the methods described, we did not detect cellulolytic or proteolytic activity of either strains. The *Pantoea* strains tested were also negative in indole and HCN production (Table 7). Phosphate solubilization, production of siderophores and IAA appear to be plant growth-promoting traits that are widespread among *Pantoea* species. For example, these activities were determined experimentally for *P. agglomerans* C1^31^, *P. eucalypti*^66^, *P. brenneri*^67^, *P. alhagi*^68^ and *P. phytobeneficialis* MSR2^9^.

**Table 7:** In vitro plant growth promoting and antimicrobial characteristics of *Pantoea* sp. MMK2^T^ and MMK3.

Characteristics	*Pantoea* sp. MMK2^T^	*Pantoea* sp. MMK3
IAA production	+	−
Indole production	−	−
HCN production	−	−
Phosphate solubilisation	+	+
Siderophore production	+	+
Cellulase production	−	−
Proteolytic activity	−	−
Antibacterial activity (growth inhibition)		
* X. vesicatoria* NCCB 92,059	+	−
* A. fabrum* C58	−	−
* P. syringae* pv. *syringae* 2905	+	−
* E. amylovora* 659	−	−
Antifungal activity (growth inhibition)		
* D. rudis* CBS 109,492	−	−
* F. oxysporum* 10Fo01	−	−
* B. cinerea* 10Bc01	−	−
* S. sclerotiorum* 10Ss01	+	+

+ activity present; − activity absent.

Analysis of the in vitro antimicrobial activity of the MMK2^T^ and MMK3 strains showed that they differ in their antifungal and antibacterial properties (Table 7). It was found that the MMK2^T^ strain inhibits growth of *X. vesicatoria* NCCB 92,059 and *P. syringae* pv. *syringae* 2905. Both MMK2^T^ and MMK3 strains have antifungal activity against the *S. sclerotiorum* strain 10Ss01. Some *Pantoea* strains are known to produce metabolites with antibacterial and antifungal activity, and biosynthetic gene clusters related to the biosynthesis of these compounds have been identified in their genomes^69^. No biosynthetic gene cluster associated with synthesis of antibiotics was identified in the genomes of studied *Pantoea* strains, suggesting another mechanism related to their antimicrobial activity. We found that the MMK2^T^ and MMK3 genomes contain genes encoding a complete type VI secretion system (T6SS), which can play different functions in pathogenic and non-pathogenic bacteria. One such role is to deliver effectors to neighbouring bacteria, inhibiting their growth and thus playing a role in interbacterial competition^70,71^. It was demonstrated that T6SS is a functional antibacterial system used by *P. agglomerans* bv. *betae* to deliver a lysozyme-like effector to eliminate competitors^72^. Further studies are required to determine the function of the T6SS in strains MMK2^T^ and MMK3.

## Conclusion

Based on the results obtained from the study of phenotypic and chemotaxonomic features as well as phylogenetic and phylogenomic analyses, the MMK2^T^ strain should be considered a novel species of the genus *Pantoea*, for which we propose the name *Pantoea trifolii* sp. nov. The protologue description of this novel species is presented in Table 8.

**Table 8:** Protologue description of *Pantoea trifolii* sp. nov.

Species name	*Pantoea trifolii*
Species epithet	*trifolii*
Species status	sp. nov
Species etymology	tri.fo’li.i. L. neut. n. *trifolium*, clover, trefoil, and also the generic name of clover (*Trifolium*); N.L. gen. neut. n. *trifolii*, of clover, of *Trifolium*
Nature of the type material	Strain
Country of origin	Poland
Date of isolation	June 2019
Source of isolation	Root nodules of *Trifolium rubens*
Sampling date	June 2019
Latitude	51.27454 N
Longitude	23.35748 E
16S rRNA gene accession	OQ799602
Genome accession number	Genome assembly accession GCA_024506435.1 Genbank accession JANIET000000000.1
Genome status	Incomplete
Genome size	5059.26 kbp
GC mol %	54.63
Number of strains in study	2
Source of isolation of non-type strains	Root nodules of *Trifolium rubens*
Designation of the Type Strain	MMK2^T^
Strain Collection Numbers	DSM 115063^T^, LMG 33049^T^
Gram staining and cell shape	Negative, rod
Colony morphology	Colonies on YEM agar are yellow in colour, size 1–2 mm in diameter after 1–2 days incubation at 28 °C
Positive tests with BIOLOG	Dextrin, d-maltose, d-trehalose, d-cellobiose, gentiobiose, β-methyl-d-glucoside, d-salicin, *N*-acetyl-d-gucosamine, *N*-acetyl-β-d-mannosamine, α-d-glucose, d-mannose, d-fructose, d-galactose, 3-methyl glucose, d-fucose, l-fucose, l-rhamnose, inosine, d-sorbitol, d-mannitol, d-arbitol, myo-inositol, glycerol, d-glucose-6-PO_4_, d-fructose-6-PO_4_, d-galacturonic acid, L-galactonic acid lactone, d-gluconic acid, d-glucuronic acid, glucuronamide, mucic acid and d-saccharic acid, l-lactic acid, citric acid, d-malic acid, l-malic acid, bromo-succinic acid, γ-amino-butyric acid, acetoacetic acid, acetic acid and formic acid. The strain also used glycyl-l-proline, l-alanine, l-arginine, l-aspartic acid, l-glutamic acid, l-histidine and l-serine
Negative tests with BIOLOG	d-turanose, stachyose, d-raffinose, α-d-lactose, d-mellibiose, *N*-acetyl neuraminic acid, d-aspartic acid, d-serine, gelatin, l-pyroglutamic acid, pectin, quinic acid, *p*-hydroxy-phenylacetic acid, methyl pyruvate, d-lactic acid methyl ester, α-keto-glutaric acid, tween 40, α-hydroksy-butyric acid, α-d,l-butyric acid, propionic acid, and minocycline
Positive enzyme activities	Alkaline phosphatase, esterase (C4), esterase lipase (C8), acid phospatase, naphthol-AS BI-phosphohydrolase, β-galactosidase, β-glucosidase and *N*-acetyl-β-glucosaminidase but not for lipase (C14), leucine arylamidase, valine arylamidase, cystine arylamidase, trypsin, α-chymotripsin, α-galactosidase, β-glucuronidase, α-glucosidase, α-mannosidase or α-fucosidase
Major fatty acids	The major cellular fatty acids are C_16:0_ (44%), C_17:0_ cyclo (17%), and C_14:0_ 3-OH (16%)

## Data Availability

This article includes all the data generated or analyzed as part of this study. The strain MMK2^T^ has been deposited in two bacterial collections: BCCM/LMG (LMG 33,049) and DSMZ (DSM 115,063). The MMK2^T^ genome sequence has been deposited in NCBI under the accession number GCA_024506435.1 (genome assembly accession) and JANIET000000000.1 (Genbank accession). The 16S rDNA gene sequences of strains MMK2^T^ and MMK3 have been deposited in NCBI under accession numbers OQ799602 and OQ799603.
